# Machine Learning in Health Care: A Critical Appraisal of Challenges and Opportunities

**DOI:** 10.5334/egems.287

**Published:** 2019-01-24

**Authors:** Mark Sendak, Michael Gao, Marshall Nichols, Anthony Lin, Suresh Balu

**Affiliations:** 1 Duke Institute for Health Innovation, US; 2 Duke University School of Medicine, US

**Keywords:** machine learning, data science, information technology, translational research, learning health

## Abstract

Examples of fully integrated machine learning models that drive clinical care are rare. Despite major advances in the development of methodologies that outperform clinical experts and growing prominence of machine learning in mainstream medical literature, major challenges remain. At Duke Health, we are in our fourth year developing, piloting, and implementing machine learning technologies in clinical care. To advance the translation of machine learning into clinical care, health system leaders must address barriers to progress and make strategic investments necessary to bring health care into a new digital age. Machine learning can improve clinical workflows in subtle ways that are distinct from how statistics has shaped medicine. However, most machine learning research occurs in siloes, and there are important, unresolved questions about how to retrain and validate models post-deployment. Academic medical centers that cultivate and value transdisciplinary collaboration are ideally suited to integrate machine learning in clinical care. Along with fostering collaborative environments, health system leaders must invest in developing new capabilities within the workforce and technology infrastructure beyond standard electronic health records. Now is the opportunity to break down barriers and achieve scalable growth in the number of high-impact collaborations between clinical researchers and machine learning experts to transform clinical care.

## Introduction

Despite excitement surrounding machine learning in health care, health systems that integrate machine learning models into clinical care are the exception rather than the rule. Bringing machine learning models from equations derived on a blackboard to care at the bedside requires intense transdisciplinary collaboration, alignment of goals, and capabilities that are hard to find in health care today. At Duke Health, we are in our fourth year developing, piloting, and implementing machine learning technologies in clinical care. To benefit from the full potential of machine learning in health care, we must acknowledge breakthroughs in technology and adoption, address barriers to progress, and critically reflect on the strategic priorities necessary to bring health care into a new digital age.

## The Good

In the last year, machine learning methods were prominently featured in mainstream medical literature. *JAMA* alone presented three deep learning models that classified images of retinopathy and breast cancer metastases at a level equal to or better than clinical experts [1,2,3]. Using tens of millions of data points from our electronic health record (EHR), our transdisciplinary team developed a deep learning model to predict onset of sepsis [4]. Well-developed models demonstrate diagnostic acumen that surpasses human capabilities and do so at scale.

Although small in number, there are emerging uses of machine learning in health care operations. For example, Epic Systems Corporation’s cognitive computing platform supports machine learning models [5]; the Food and Drug Administration has approved software to assist with medical imaging segmentation [6]; and patient deterioration models are commonly built into EHRs. The adoption of such models and technologies serves as a foundation for machine learning to diffuse across institutions into clinical care operations.

## The Bad

Historically, statistical models in health care found patterns in data that enhanced clinical reasoning. This expectation is often applied to machine learning models, but machine learning and clinical reasoning are not always coupled. Clinical reasoning is often cultivated across institutions, while machine learning models are often developed using data from a single institution and have limited generalizability. For example, a *Clostridium difficile* model tested at two academic medical centers revealed variables that were top risk factors in one setting and protective in the other [7]. Clinical care processes that generate and capture data vary widely across institutions and local biases are baked into machine learning models. However, even if a model cannot enhance clinical reasoning, it can still augment workflow-specific, local decisions.

If health system leaders want to test a newly validated machine learning model in their local environment, they must prepare for significant investment in personnel and technology. Culling through raw health care data to construct meaningful features is expensive and time-intensive. At our institution, the cost of developing, validating, and integrating a single analytics tool to identify patients at high-risk of dialysis was $220,000 [8]. At a national level, the cost of abstracting and normalizing data captured in the EHR to report quality measures is $15.4 billion [9]. Resource requirements prevent even the most generalizable model from efficiently scaling across institutions.

Almost all research at the intersection of machine learning and health care is performed on remotely collected, stale data without appropriate transdisciplinary domain expertise. During 2015–2017, the *Journal of Machine Learning Research* had three issues dedicated to health care, including a special feature and two proceedings for the “Machine Learning in Healthcare Conference.” Of 40 publications, 23 (57.5 percent) had a clinical collaborator, 10 (25 percent) used non-MIMIC (Medical Information Mart for Intensive Care) [10] EHR data, and only seven (17.5 percent) had both a clinical collaborator and used locally collected, non-MIMIC EHR data (see Appendix 1: Supplemental Data). Three of the seven papers were projects our group worked on, and all seven were from academic medical centers with quantitative sciences and clinical departments, including New York University; University of California, San Diego; and University of Southern California. Without engaging partners across domains to solve relevant, local problems, machine learning will continue to struggle with adoption by both clinicians and health information technology leaders.

## The Ugly

Personalized medicine will require mass customization of models that are trained and re-calibrated at the hospital and cohort level. Modern machine learning techniques focus on generalization beyond a training dataset, not on generalization to different sites. Transfer learning methods require further development to help address this problem, and in the meantime generalization must be achieved through localization. Adapting a model to a local setting requires either the skills to extract and curate local datasets and retrain models at every site or a willingness to leverage capabilities from outside institutions.

Methods for evaluating and monitoring models to ensure continued accuracy and performance are in their infancy. The underlying data structure of EHRs is highly dynamic and can result in errors when models are evaluated. For example, a new medication, order set in the EHR, or blood chemistry analyzer in the lab can change metadata and cause downstream errors in data processing. Machine learning models and infrastructure need to account for these changes so that their results are robust to the underlying conditions. In addition, although machine learning has developed methods for model validation, such as training-test-validation splits and k-fold cross-validation [11], further validation of a model post-implementation requires new techniques. Consider the case of a machine learning model used to predict the onset of sepsis. If action taken as a result of the model prevents infection, the counterfactual to this event is not observed and there is no clear way to classify the event as a false positive or a successful intervention. Compounding this issue, if the model is retrained at a later time using data from the post-implementation period, the results can be biased in ways that are difficult to ascertain. New methods and technology infrastructure must be developed to address these complex issues.

## The Opportunity

Institutions that are interested in embedding machine learning into clinical care operations must coalesce a workforce with new competencies, harness transdisciplinary resources, and invest in platforms to support machine learning. In 2015, Thomas Davenport and Julia Kirby characterized five ways knowledge workers can respond to automation [12]. Applying their framework to health care, providers can *step up* (consider the big picture of the industry), *step aside* (develop strengths that aren’t codifiable cognition), *step in* (modify and monitor software), *step narrowly* (specialize in something for which no computer program has yet been developed), or *step forward* (build the next generation of technology). At worst, financial and cultural pressures drive clinicians to *step narrowly* to specialize and hide from technology. At best, informatics and statistics training drive clinicians to *step in* to modify and monitor software. If clinicians are to *step forward*, health system leaders must invest in programs that empower the clinical workforce to develop next-generation technologies. This requires health system leaders to shift from viewing technology development as an expense to viewing technology development as an investment in future growth.

Academic medical centers that cultivate and value transdisciplinary collaborations and training are ideally suited to embed machine learning in routine clinical care. At Duke Health, we embed statistics and computer science students with medical students on teams led by clinical and quantitative science experts. Between 2015–2017, we trained 40 undergraduate and masters students studying statistics and computer science, 2 doctoral students studying statistics, and eight medical student research scholars. Our medical student scholars have presented 18 abstracts at research conferences, published 9 papers [13,14], operationalized 7 technology products within our health care system, and disclosed 6 inventions to our technology transfer office [15]. Through this process, we have coupled career development with successful pilot implementations to improve clinical care.

The time has come for health system leaders to refocus attention and energy from siloed applications of machine learning in health care to the underlying platforms required to efficiently scale machine learning across health care. At Duke Health and as an industry, we have witnessed a critical mass of successful projects and collaborations. Now is the opportunity to reflect on learnings and expose and break down barriers to build platforms that support many machine learning applications. Health system leaders must redefine success from optimizing performance metrics of a single model to optimizing scalable growth in the number of high-impact collaborations between clinical researchers and machine learning experts.

## Additional File

The additional file for this article can be found as follows:

10.5334/egems.287.s1 Appendix 1. Supplemental Data.
